# Synovial Fluid in Knee Osteoarthritis Extends Proinflammatory Niche for Macrophage Polarization

**DOI:** 10.3390/cells11244115

**Published:** 2022-12-18

**Authors:** Priya Kulkarni, Vanshika Srivastava, Kaspar Tootsi, Ali Electricwala, Avinash Kharat, Ramesh Bhonde, Sulev Koks, Aare Martson, Abhay Harsulkar

**Affiliations:** 1 Department of Pathophysiology, Institute of Biomedicine and Translational Medicine, University of Tartu, Ravila 19, 50411 Tartu, Estonia; 2 Department of Traumatology and Orthopaedics, Institute of Clinical Medicine, University of Tartu, L Puusepa 8, 51014 Tartu, Estonia; 3 Department of Pharmaceutical Biotechnology, Poona College of Pharmacy, and Interactive Research Schools for Health Affairs, Bharati Vidyapeeth Deemed to be University, Erandwane, Pune 411038, India; 4 Electricwala Hospital, A 4/1, Pleasant Park, Fatima Nagar, Wanowrie, Pune 411013, India; 5 Regenerative Medicine Laboratory, Dr. D. Y. Patil Dental College & Hospital, Dr. D. Y. Patil Vidyapeeth, Pune 411018, India; 6 Perron Institute for Neurological and Translational Science, Nedlands, WA 6009, Australia; 7 Centre for Molecular Medicine and Innovative Therapeutics, Murdoch University, Murdoch, WA 6150, Australia; 8 Clinic of Traumatology and Orthopaedics, Tartu University Hospital, L Puusepa 8, 51014 Tartu, Estonia

**Keywords:** Kellgren-Lawrence score, macrophages, M1/M2 ratio, niche, osteoarthritis, synovial fluid, synovitis

## Abstract

Macrophage polarization is a steering factor of osteoarthritis (OA) progression. Synovial fluid (SF) obtained from OA patients with different Kellgren–Lawrence grades (KL grades) holds several proinflammatory factors and was hypothesized to induce macrophage differentiation and polarization by providing the needed microenvironment. U937 cells and peripheral-blood-mononuclear-cell-derived monocytes (PBMC-derived CD14+ cells) were induced with SFs of progressive KL grades for 48 h, and the status of the differentiated cells was evaluated by cell surface markers representing M1 and M2 macrophage phenotypes. Functional viability assessment of the differentiated cells was performed by cytokine estimation. The fraction of macrophages and their phenotypes were estimated by immunophenotyping of SF-isolated cells of different KL grades. A grade-wise proteome analysis of SFs was performed in search of the factors which are influential in macrophage differentiation and polarization. In the assay on U937 cells, induction with SF of KL grade III and IV showed a significant increase in M1 type (CD86+). The percentage of M2 phenotype (CD163+) was significantly higher after the induction with SF of KL grade II. A Significantly higher M1/M2 ratio was estimated in the cells induced with KL grade III and IV. The cell differentiation pattern in the assay on PBMC-derived CD14+ cells showed a grade-wise decline in both M1 (CD11C+, CD86+) and M2 phenotype (CD163+). Cytokine estimation specific to M1 (TNF-α, IL-6, IL-1β, IFN-γ) and M2 (IL-4 and IL-10) macrophages corelated with the differentiation pattern in the U937 cell assay, while it did not reveal any significant changes in the PBMC-derived CD14+ cells assay. SF cells’ immunophenotyping showed the highest percentage of CD14+ macrophages in KL grade II; CD86+ and CD163+ cells were minimal in all KL grades’ SFs. The proteome analysis revealed significantly expressed MIF, CAPG/MCP, osteopontin, and RAS-related RAB proteins in KL grade III and IV samples, which are linked with macrophages’ movement, polarization, and migration-behavior. In conclusion, this study demonstrated that SF in OA joints acts as a niche and facilitates M1 phenotype polarization by providing a proinflammatory microenvironment.

## 1. Introduction

Osteoarthritis (OA) is one of the most debilitating musculoskeletal disorders that exists in the elderly population. The conventional understanding of OA being an aging disorder has been challenged in recent times, and a growing body of evidence presents synovial inflammation as a driving pathology for the symptomatic and structural progression of OA. A significant macrophage infiltration in the intimal lining of the synovium is a hallmark of synovitis in OA; the cells are known to play a critical role in the pathology by implicating inflammatory and destructive responses [1,2,3].

Macrophages possess a strong plasticity [4], and based on the stimuli, they are broadly categorized into two sub-groups—M1 and M2 types. M1 macrophages are proinflammatory and release a large number of proinflammatory cytokines, including interleukin-1β (IL-1β), tumor necrosis factor-α (TNF-α), IL-12, IL-6, chemokines (CXCL11, CXCL9 and CXCL10), and MMP-1, 2, 7, 9, and 12. In contrast, M2 macrophages resolve inflammation, contribute to tissue homeostasis, and release anti-inflammatory cytokines (IL-10 and IL-4), chemokines (CCL17), and growth factors like vascular endothelial growth factor (VEGF) and transforming growth factor (TGF-β) [5]. Macrophage polarization is a steering factor of OA pathology and a failure of transformation from M1 to M2 type is thought to contribute to its development and progression [6,7,8]. Furthermore, it is well understood that the outcome of cell transformation is highly dependent on its microenvironment. On this background, the present study was initiated to investigate the potential of OA synovial fluid (SF) to induce macrophage polarization. OA SF is known to hold several proinflammatory factors, cell and matrix degradation products, and immunoglobulins. This naturally existing pathobiological cocktail can induce cellular inflammation, as shown by the authors in their previous published studies [9,10]. In a similar vein, SF was shown to stimulate metabolic activity in SF-isolated cells [11]. SF obtained from patients with aseptic prosthesis loosening had a stimulatory effect on collagen formation and induced cell proliferation in MG63 osteoblasts [12]. Bovine SF in low concentration was shown to stimulate proliferation in rabbit medial cruciate ligament and anterior cruciate ligament, while its higher concentrations did not cause any inhibitory effect on ligament proliferation [13]. Thus, it can be interpreted that SF proteins, oxidant radicals, tissue degradation products, systemic signals, and SF-resident cells contribute to the inducing potential of SF, influencing a spectrum of joint cells, and are likely to modulate their function. Using SFs from the affected patients for in vitro testing is therefore an attractive model to study chronic disease pathologies like OA.

In the present study, using U937 and peripheral-blood-mononuclear-cell-derived monocytes (PBMC-derived CD14+ cells), we show that SFs from OA patients of different severity act as a niche and induce macrophage differentiation and polarization by providing the necessary microenvironment. We also present an account on the functional status of the newly differentiated cells.

## 2. Methods

### 2.1. Cell Lines

A human-monocyte-like cell line (U937) was purchased from National Centre for Cell Science (NCCS), Pune, India. In addition, peripheral blood mononuclear cells (PBMCs) were freshly isolated using the blood of healthy donors. The PBMCs’ isolation process is described later in this section. Both cell types were maintained in Roswell Park Memorial Institute (RPMI) 1640 medium (HiMedia Laboratories, LLC, Kennett Square, PA, USA) + 10% Fetal Bovine Serum (FBS) (HiMedia Laboratories, LLC, Kennett Square, PA, USA) + 2 mmol/L L-glutamine + 1% penicillin + streptomycin (Sigma-Aldrich, St. Louis, MO, USA) at 95% relative humidity and 5% CO_2_ at 37 °C.

### 2.2. SF Collection

The SF samples used in the various experiments of this study were obtained from OA patients with a confirmed disease diagnosis (age group—40 to 75 years) at the Bharati hospital and research center, Pune and Electricwala hospital and joint replacement center, Pune, India. OA diagnosis was performed by experienced rheumatologist/orthopedic surgeon based on clinical signs, symptoms, and radiographic features. Severity of OA was determined by Kellgren–Lawrence (KL) radiographic score, which is based on the radiological signs as described elsewhere [14]. Grade I is doubtful narrowing of the joint space and possible, indistinguishable osteophytes; grade II is definite clearly identifiable osteophytes and possible narrowing of the joint space; grade III is with moderate multiple osteophytes with definite joint space narrowing; and grade IV is marked with large osteophytes with marked narrowing of joint space. Patients with rheumatoid arthritis (RA), infected arthritis, OA with tumors, or OA with a history of knee joint injuries in the past 2 years were excluded.

In total, twenty SF samples spanning different KL scores were collected by knee arthrocentesis (n = 20); it included five SF samples of each KL grade (5 × 4 = 20). Demographic details of the patients from whom the SF samples were obtained are provided in Table S1 of Supplementary Data. The knee arthrocentesis was performed as described [15]. For collection of KL grade, I, II, and III samples, the arthrocentesis was performed when the patient had a knee effusion and was required to undergo the procedure to relieve the disease symptoms. This procedure took place in a minor operation theater under strict aseptic conditions. The samples of KL grade IV were collected at the time of knee replacement surgery. SF sample grading was the same as OA grading. All the recruited patients signed an informed written consent form before participating in the study.

### 2.3. Immunophenotyping of SF Cells

**Figure 1:**
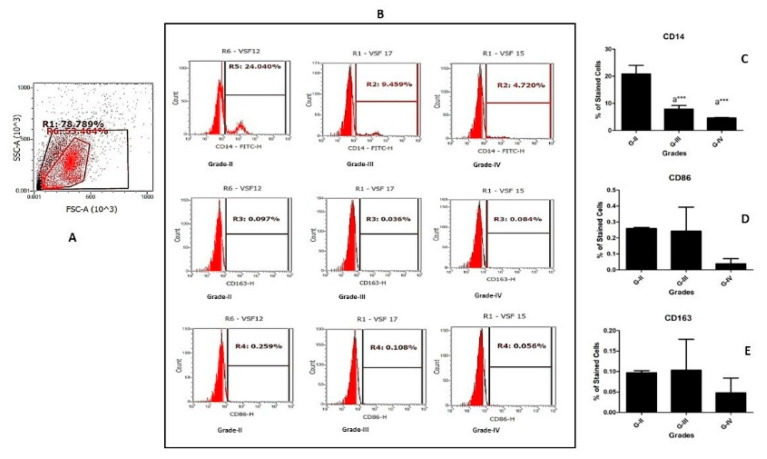
Immunophenotyping of SF samples. Twelve freshly collected SF samples were subjected to immunophenotyping to estimate monocyte/macrophage percentage. (**A**) denotes the gating strategy established from isotype control for the selection of cell population. (**B**) shows representative histograms of macrophages and their subset analysis in OA SFs from KL grade II, III, and IV; for the quantification of macrophages and their subsets, the used antibody panel included cell surface markers like CD14 (pan-macrophage marker), while CD86 and CD163 represent M1 and M2 phenotype of macrophages, respectively. Values in the quadrant represent the percentage of positive cells. (**C**–**E**) are the bar graphs, representing a grade-wise staining pattern of CD14+, CD86+, and CD163+ cells in SF samples; for the graph plotting, the average of each cell surface marker obtained from three SF samples of each KL grade was used (n = 12). **^a^** statistical significance in comparison to G-II; *** *p* < 0.001.

Immunophenotyping of SF cells from twelve samples (four SF samples from each KL grade II, III, and IV) was carried out to determine the percentage of macrophages in the fluids. These fluids were further used to induce U937 and PBMC-derived CD14+ cells, as explained later. For this, the collected samples were processed within 24 h of collection time. After the collection, the samples were carefully examined for color, opacity, viscosity, and any blood contamination. The samples with any sign of blood contamination were excluded. Additionally, an aliquot of each sample was submitted for microbial examination. For isolation of cells in SF, the fluids were centrifuged at 1200 rpm at 4 °C for 20 min. The obtained cell pallets were twice washed with ice-cold phosphate buffer saline (PBS) at 1200 rpm at 4 °C for 5 min to remove any traces of SF. The resulting cell pallet was aliquoted and used to stain with macrophage-specific-fluorescent-labelled antibodies, including CD14 (FITC, BioLegend, San Diego, CA, USA), CD86 (PECy7, BioLegend, San Diego, CA, USA), and CD163 (PerCP 5.5, BioLegend, San Diego, CA, USA) as per the manufacturer’s instructions. The stained cells were acquired using a gating strategy as indicated in Figure 1 (based on isotype control) and further analyzed on Attune Nxt Acoustic flow cytometer (Thermofisher Scientific, Waltham, MA, USA). For each experiment reaction, 10,000 events were acquired. The flow cytometry data analysis was performed using Attune Nxt software (version 2.1), and the results are expressed as the percentage of positively stained cells.

### 2.4. In Vitro Cell Differentiation Assay on U937 Cells

This assay was designed to assess the potential of OA SF in inducing immune cell differentiation and polarization. In the assay, U937 cells were treated as immune cell precursors and were exposed to different grades of OA SFs for 48 h. After the incubation period, the status of the newly differentiated cells was analyzed using relevant flow cytometry markers. The assay protocol was as follows –

U937 cells were seeded with a density of 1 × 10^6^ cells/mL in a 24-well plate and were induced with 20% (of culture medium) SF of different OA grades for 48 h. We used four samples of each KL grade II, III, and IV for inducing the differentiation; in total, twelve OA SF samples were used. The cells induced with phorbol 12-myristate 13-acetate (PMA) (Sigma-Aldrich, St. Louis, MO, USA) (dose—100 ng/mL) were used as a positive control, and the untreated cells were used as a negative control. A safe dose of PMA for the induction was determined by cell viability assay, which was performed as described before [16]. After the incubation period, adherent cells were collected for flow cytometry analysis. To harvest the cells without enzymatic digestion, they were incubated in 0.5 mM ethylenediamine tetra-acetic acid (EDTA) in PBS for 15 min at 37 °C and 5% CO_2_. After 15 min, the cells were collected by repeated vigorous pipetting against the bottom of the 24-well plate. The harvested cells were first blocked with human FC blocking solution (Human TrueStain FcX, BioLegend, San Diego, CA, USA) to prevent any non-specific antibody binding and further stained with CD14 (FITC, BioLegend, San Diego, CA, USA), CD86 (PECy7, BioLegend, San Diego, CA, USA) and CD163 (PerCP 5.5, BioLegend, San Diego, CA, USA) as per the manufacturer’s instructions. For each marker, positively stained cells were acquired using BD FACSCanto II cell analyzer (BD Biosciences), and 10,000 events were counted. The data analysis was performed with FACSDiva software (version 6.1.2). These flow cytometry experiments were performed in quadruplets and repeated three times; the results are presented as a percentage of positively stained cells for each cell surface antibody.

### 2.5. Functional Analysis of the Newly Differentiated Cells in U937 Cell Assay

The flow cytometry analysis was followed by an assessment of the functional status of the newly differentiated cells after 48 h in the U937 cell assay in terms of the estimation of cytokine expressions produced by these cells. For this, total RNA extraction was performed from the cell pallet using TRIZOL reagent (Invitrogen Co., Carlsbad, CA, USA) and later with SuperScript-III Cell to cDNA kit (Invitrogen Co., Carlsbad, CA, USA) following the manufacturer’s instructions. Total RNA was quantified by measurement of UV absorbance at 260 nm. First-strand cDNA was synthesized from 1 μg of total RNA using the SuperScript First-Strand Synthesis System for qRT-PCR (Invitrogen Co., Carlsbad, CA, USA). qRT-PCR analysis was performed with Applied Biosystems’ StepOne Real Time PCR System using TaqMan gene expression assays (TNF-α: Hs00174128_m1, IL-6: Hs00985639_m1, IFN-γ: Hs00989291_m1, GMCSF: Hs00929873_m1, IL-4: Hs00174122_m1, IL-10: Hs00961622_m1, and ACTB: Hs01060665_m1, Applied Biosystems, Foster City, CA, USA) and TaqMan Gene Expression Master Mix (Applied Biosystems, Foster City, CA, USA). mRNA expressions were normalized against the house keeping gene β-actin, while analysis was done using Step One Software version 2.2.2.

### 2.6. Blood Collection for PBMC Isolation

The in vitro cell differentiation assay was further repeated on PBMC-derived CD14+ cells. For this, first, fresh PBMCs were isolated from human whole blood and the cells were further subjected to cell sorting to obtain CD14+ cells. For this assay, about 7–10 mL of blood was obtained from each healthy donor (age group—40 to 70 years) at Bharati Hospital, Pune, India. A total of six healthy donors were recruited for this study; each donor was briefed about the purpose of the study and written informed consent was obtained. The sample collection process was performed by a trained laboratory technician under aseptic conditions; the blood sample was collected in a heparin-coated sterile bulb and immediately carried at RT for PBMC isolation.

### 2.7. PBMC Isolation

The collected blood samples were subjected to PBMC isolation using SepMate™-15 PBMC Isolation Tubes (Stem Cell Technologies, Vancouver, BC, Canada) as per the manufacturer’s instructions. In brief, the bottom part of the SepMate™-15 PBMC Isolation Tube was filled with 2.5 mL of histopaque-1077 density gradient (Sigma-Aldrich, St. Louis, MO, USA); the collected blood samples were diluted with equal volume of PBS, and approximately 10 mL of the diluted blood sample were carefully placed on the top part of SepMate™-15 PBMC Isolation Tube. The PBMC isolation tubes were subjected to centrifugation at RT at 1200 g for 20 min. The upper layer of the PBMC isolation tube was poured into a 15 mL Falcon tube. Isolated PBMCs were washed twice with DPBS (PBS + 2% FBS) and were immediately used for cell sorting.

### 2.8. Cells Sorting

Freshly isolated PBMCs were stained with CD14 cell surface marker (FITC, BioLegend, San Diego, CA, USA) as per the manufacturer’s instructions. CD14+ cell sorting was performed on BD ARIA II SORP. BD ARIA II SORP is a special-order research product with 4 lasers (blue 488 nm, red 640 nm, violet 405 nm, and UV 355 nm) and 11 colors. While cell sorting, the gating strategy was based on isotype control as indicated in Figure S1 of Supplementary Data. The sorted CD14+ cells were collected in a 15 mL Falcon tube containing cell culture media (RPMI-1640 + 10% FBS + 1% penstrep) and carried on ice for further processing.

### 2.9. In Vitro Cell Differentiation Assay on PBMC-Derived CD14+ Cells

The in vitro cell differentiation assay on PBMC-derived CD14+ cells using the same protocol as the assay on the U937 cells. Considering the limited number of sorted cells, this assay was performed using early and end-stage SF samples only. Thus, PBMC-derived CD14+ cells were induced using SFs of KL grade II and IV. After 48 h of SF treatment, the induced cells were analyzed using a flow cytometry panel including cell surface markers like CD11C (FITC, BioLegend, San Diego, CA, USA), CD86 (PECy7, BioLegend, San Diego, CA, USA), and CD163 (PerCP 5.5, BioLegend, San Diego, CA, USA) as per the manufacturer’s instructions. The cell acquisition and data analysis were the same as the assay on the U937 cells. These flow cytometry experiments were performed in triplicate and repeated three times. In total, six OA SFs (three SFs of grade II and three SFs of grade IV) were used for this set of experiments.

### 2.10. Functional Analysis of the Newly Differentiated Cells in the Assay on PBMC-Derived CD14+ Cells

After 48 h, the activity status of the newly differentiated SF-induced PBMC-derived CD14+ cells were evaluated by measuring the cytokines produced by the differentiated cells. Because of a limited number of sorted cells, the authors were unable to perform the cytokine estimation in the form of a gene expression assay. Instead, the cytokine measurement was performed using LEGENDplex™ HU Essential Immune Response Panel (13-plex) (BioLegend, San Diego, CA, USA; Catalogue No. 740930) as per the manufacturer’s instructions. Mean fluorescence intensity (MFI) of each cytokine in the panel was determined using the standard curve provided in the assay kit.

### 2.11. Proteome Analysis of OA SF Samples

Proteome analysis of the SF samples, which were used for the cell induction during in vitro assays, was performed in search of the proteins that could drive or regulate monocyte/macrophage differentiation and polarization. Sixteen SF samples, wherein we included four samples of each KL grade, were subjected to the analysis (4 × 4 = 16). At first, a protein depletion column was used to deplete abundantly present albumin and immunoglobulins from the samples as per the manufacturer’s instruction (BioRad proeteoMinerprotein enrichment small-capacity kit; catalogue No. 1633006). Protein precipitation was achieved by mixing each SF sample with a mixture of 100% trichloroacetic acid and 0.4% deoxycholate (TCA + DOC) in a 1:3 (*v*/*v*) ratio for 20 min at 4 °C and later centrifuging for 15 min at 17,000 rpm. The supernatant was discarded; the undisturbed pellet was resuspended in 3 volumes (of the original sample volume) of RT 100% acetone, vortexed, incubated for 10 min at RT, and centrifuged at 17,000 rpm for 15 min. All the precipitates were air dried on ice for 10 min until no residual liquid was visible, and the precipitated pellets were further suspended in 100 μL of 7 M urea, 2 M thiourea, and 100 mM ammonium bicarbonate (ABC) solution (7/2 urea:thiourea buffer). Reduction and alkylation of cysteine bonds was achieved by mixing each sample with 5 mM dithiothreitol and 20 mM chloroacetamide, respectively, for 1 h at RT in the dark. Furthermore, each sample was digested for 4 h in a 1:50 (enzyme: protein) ratio using *Lysobacter enzymogenes* (Wako Pure Chemical Industries, Richmond, VA, USA). Each sample solution was diluted five times with 100 mM ABC and further digested overnight at RT with 1:50 dimethylated porcine trypsin (Sigma, Aldrich). All the digested samples were then desalted using reversed-phase C18 StageTips. Lastly, the samples were reconstituted in 0.5% trifluoro acetic acid (TFA) for the subsequent LC/MS/MS analysis, which was performed as described [17].

The raw data of MS were processed with the MaxQuant 1.4.0.8 software package [18]. Methionine oxidation, asparagine/glutamine deamidation, and protein N-terminal acetylation were set as variable modifications, while cysteine carbamidomethylation was defined as a fixed modification. Peptide search was conducted against in silico trypsin digested (C-terminal cleavage after lysine/arginine without proline restriction) in the UniProt (access date – 13 November 2020; 19, UniProt Consortium, Cambridge, UK) *Homo sapiens* reference proteome database [19]. First and main search MS mass tolerances were ±20 and ±4.5 ppm, respectively. MS/MS mass accuracy tolerance was ±20 ppm. Protein identifications were reported if ≥1 razor or unique peptides of ≥7 amino acids were known. Transfer of peptide identifications (match between runs) based on accurate MS1 mass and RT was allowed. Protein quantification was reported if ≥1 peptide was quantified with ≥3 points. Label-free protein intensities were normalized using the MaxLFQ algorithm [20]. Peptide spectrum match and protein false discovery rate (FDR) were kept ≤1% using a target-decoy approach. All other parameters were set as default.

### 2.12. Statistical Analysis

Data analysis of the in vitro cell differentiation assay on U937 and PBMC-derived CD14+ cells was done using FACSDiva software (version 6.1.2); data analysis of SF immunotyping was performed using attune Nxt software (version 2.1). qRT-PCR data analysis and inter-grade comparison were performed by one-way ANOVA followed by Bonferroni’s multiple comparison test using the GraphPad Prism 5 Program (San Diego, CA, USA). In the proteome analysis, the protein values of KL grade I SF samples were used as control and comparative fold change values of differentially expressed proteins in the KL grade II, III, and IV samples were presented after performing a paired *t*-test. In all the experiments, *p*-values < 0.05 were considered as significant.

## 3. Results

### 3.1. Immunophenotyping of SF-Isolated Cells to Estimate Immune Cell Fractions

Immunophenotyping of the cells in freshly collected SF samples was performed, focusing on the estimation of monocytes/macrophages. Thus, the antibody panel selected for these experiments included cell surface markers like CD14, CD86, and CD163, which denote monocytes/macrophages and their phenotypes. In these experiments, four SF samples of each KL grade II, III, and IV were included (n = 12). The percentage of CD14+ monocytes/macrophages was highest in KL grade II samples, followed by a significant decline in KL grade III and IV (Figure 1C). The percentage of CD86+ and CD163+ cells also showed a grade-wise decline; however, the percentages of these cells were minimal (Figure 1D,E).

### 3.2. In Vitro Cell Differentiation Assay on U937 Cells

**Figure 2:**
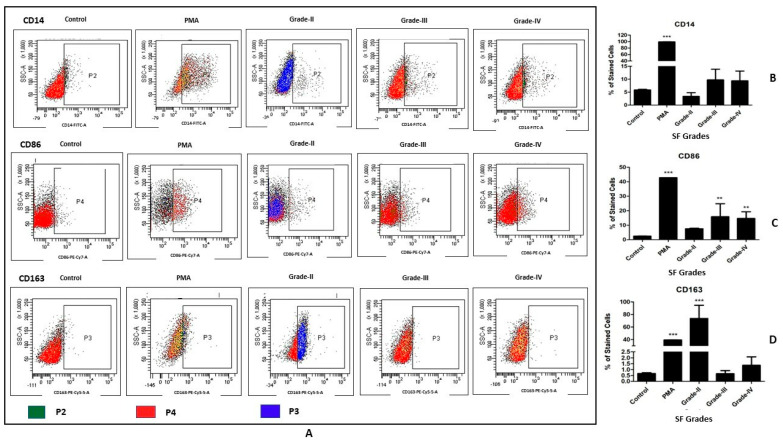
In vitro cell differentiation assay on U937 cells. U937 cells were induced using SF of KL grade II, III and IV for 48 h and status of the newly differentiated cells were evaluated using macrophage-specific cell surface markers—CD14, CD86, and CD163; (**A**) representative scatterplots of macrophages and their subtypes; CD14 is a marker for M0 macrophages, while CD86 and CD163 represent M1 and M2 subtype, respectively; the gating strategy of cell population selection was based on isotype control; (**B**–**D**) demonstrate a grade-wise staining trend for CD14+, CD86+ and CD163+ cells, respectively; for these graphs, we used the average percentage of each cell surface marker calculated from three sets of experiment, wherein four SF samples of each KL grade were used in each set of experiment (n = 12); the experiment was performed in quadruplet and repeated for three times. ** *p* < 0.01, *** *p* < 0.001, as compared to control.

The status of newly differentiated cells after 48 h of SF induction was evaluated with a macrophage-specific antibody panel including CD14, CD86, and CD163. A grade-wise increase was noted in the percentage of CD14+ cells; this means that the highest percentage of CD14+ cells was seen after SF induction with KL grade IV. Inter-grade comparison for CD14+ cell phenotype was insignificant on a statistical scale (Figure 2B). CD86+ cells also exhibited a differentiation pattern similar to CD14+ phenotype; thus, a grade-wise elevation was noted in the CD86+ phenotype. Induction with SF of KL grade III and IV showed a marked increase in the percentage of CD86+ cells (*p* < 0.01) (Figure 2C). Unlike CD14 and CD86, the percentage of CD163+ cells dramatically increased after the induction with KL grade II (*p* < 0.001). This increase was followed by a decline after the treatment of KL grade III and was comparable to the percentage of CD163+ cells in the control. KL grade IV SF treatment showed some increase in CD163+ cells (Figure 2D).

### 3.3. A Functional Status Analysis of the Newly Differentiated Cells in the Assay on U937 cells

**Figure 3:**
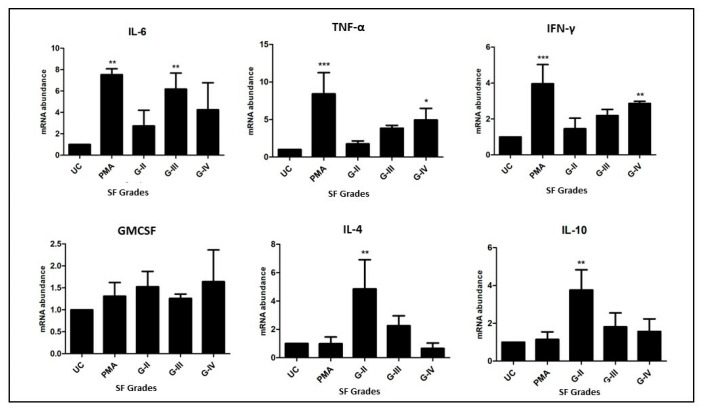
Functional status of the newly differentiated cells from in vitro U937 cells was investigated in terms of mRNA abundance of the cytokine gene produced by the new cells. The expression amount of each cytokine gene was normalized against ACTB, a house-keeping gene; in the cytokine panel, IL-6, TNF-α, IFN-γ, and GMCSF are M1-macrophage-specific, and IL-4 and IL-10 are M2-specific cytokines; * *p* < 0.05, ** *p* < 0.01, *** *p* < 0.001, as compared to UC.

In this, the cytokine gene expressions in the newly differentiated cells were measured by qRT-PCR. In the selected cytokine genes panel, IL-6, TNF-α, IFN-γ, and GMCSF are proinflammatory in nature (M1 macrophage specific), while IL-4 and IL-10 are known to be produced by the M2 type of macrophages. The expression of IL-6 was significantly higher in the differentiated cells, which were induced by SF of KL grade III, when compared to untreated cells (*p* < 0.01). TNF-α and IFN-γ expressions were noted to be significantly higher in the cells induced with SF of KL grade IV (*p* < 0.05 and *p* < 0.01, respectively). On the other hand, IL-4 and IL-10 showed a similar expression pattern with the peak in the differentiated cells, which were induced by SF of KL grade II (*p* < 0.01). The expressions of GMCSF did not reveal any specific trend. In summary, all the cytokine expressions, excluding GMCSF, were well correlated with their flow cytometry analysis (Figure 3).

### 3.4. In Vitro Cell Differentiation Assay on PBMC-Derived CD14+ Cells

**Figure 4:**
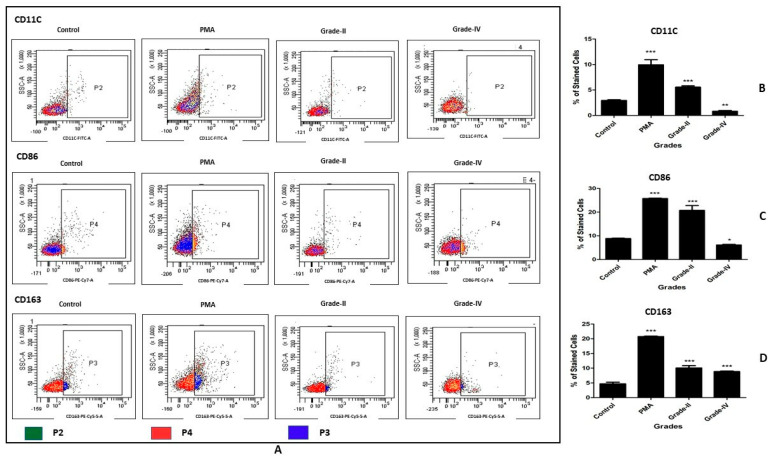
In vitro cell differentiation assay on PBMC-derived CD14+ cells. PBMC-derived CD14+ cells were induced with SF of KL grade II and IV for 48 h; the status of the newly differentiated cells was evaluated with the cell surface markers CD11C, CD86, and CD163. (**A**) shows representative scatterplots of the differentiated macrophages and their subsets; CD11C and CD86 are M1 markers, while CD163 is an M2 subtype marker; the gating was based on isotype control. (**B**–**D**) shows a grade-wise staining pattern of CD11C+, CD86+, and CD163+ cells; to create these bar graphs, the average percentage of each cell surface marker estimated from three SF samples of each KL grade was used (n = 6); the experiment was performed in triplicate and repeated three times. * *p* < 0.05, ** *p* < 0.01, *** *p* < 0.001, as compared to control.

In this assay, the SF treatment was performed using fluid samples from KL grade II and IV, and the antibody panel used for evaluation was CD11C, CD86, and CD163. The percentage of CD11C+ cells showed a grade-wise decline; the highest percentage was found in the cells induced with KL grade II SF samples, which was significantly higher as compared to untreated cells (*p* < 0.001). The treatment with KL grade IV, however, showed a significant decline in the percentage of CD11C cells when compared to untreated cells (*p* < 0.01) (Figure 4B). The differentiation pattern of CD86 was similar to CD11C, and a grade-wise decline was found in the positively stained cell percentage. The percentage of CD86+ cells was highly significant after the treatment with KL grade II as compared to control (*p* < 0.001). On the contrary, CD86+ cell percentage was marginally reduced when PBMC-derived CD+ cells were induced with KL grade IV SF samples (*p* < 0.05) (Figure 4C). The expression trend of CD86+ cells did not match the expression trend of CD86+ cells in U937, which showed a grade-wise increase. The percentage of CD163+ cells showed a grade-wise decline; the highest percentage of positively stained cells was found in the cells treated with SF samples of KL grade II (*p* < 0.001). Induction with KL grade IV also caused a significant rise in the percentage of CD163+ cells (*p* < 0.001) (Figure 4D). This differentiation trend was similar to the differentiation trend of CD163+ cells in the U937 experiment.

### 3.5. A Functional Status Analysis of the Newly Differentiated Cells in the Assay on PBMC-Derived CD14+ Cells

**Figure 5:**
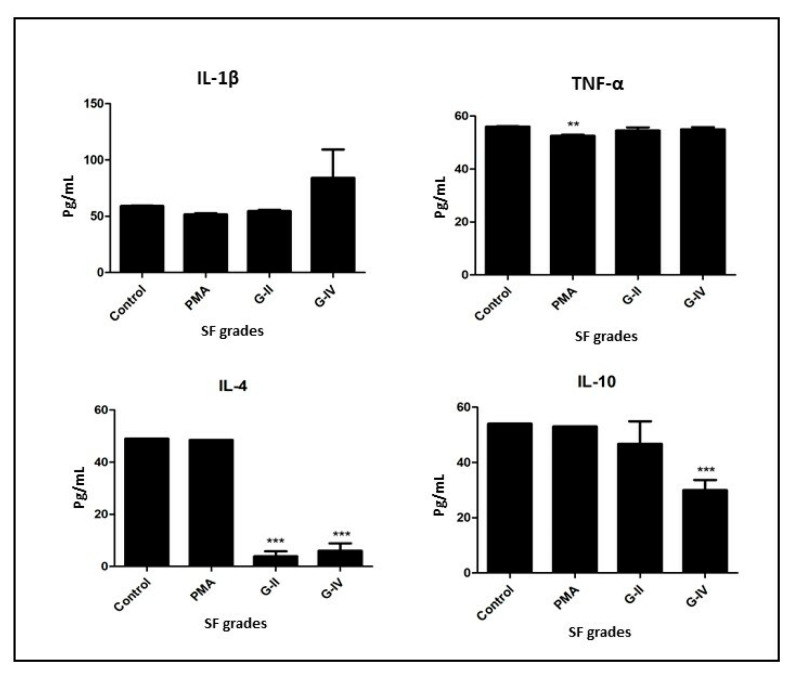
Functional status of the newly differentiated cells from in vitro PBMC-derived CD14+ cells was investigated by estimating cytokine levels in the cell culture media after 48 h of SF induction; in the figure, the X-axis denotes different KL grades of SF samples used for the induction, while the Y-axis represents the level of each selected cytokine in pg/mL; in the selected cytokines panel, IL-1β and TNF-α are M1-macrophage-specific, and IL-4 and IL-10 are M2-specific cytokines; ** *p* < 0.01, *** *p* < 0.001, as compared to control.

In this analysis, the functional ability of the newly differentiated cells was measured in terms of the level of released cytokines as estimated in the cell culture media after 48 h of SF induction. IL-1β showed comparable levels in the control, PMA, and the cells differentiated by the induction of KL grade II. An insignificant increase was noted in the level of the differentiated cells by the induction with SF of KL grade IV. Additionally, comparable levels of TNF-α were estimated in the control and the cells differentiated by the induction of KL grade II and IV SFs. The levels in the differentiated cell induced by PMA were significantly lower (*p* < 0.01) (Figure 5).

The levels of IL-4 showed a significant decline in the differentiated cells wherein the SF induction was performed with KL grade II and IV (*p* < 0.001); on the other hand, IL-10 was found to have dropped in the differentiated cells by the induction with SF of KL grade II as well as IV. This drop was significant for the induction with KL grade IV (*p* < 0.001). In summary, we did not find any notable changes in the cytokine estimation of the differentiated cells in the in vitro assay on PBMC-derived CD14+ cells (Figure 5).

### 3.6. M1/M2 Ratio Estimation

**Figure 6:**
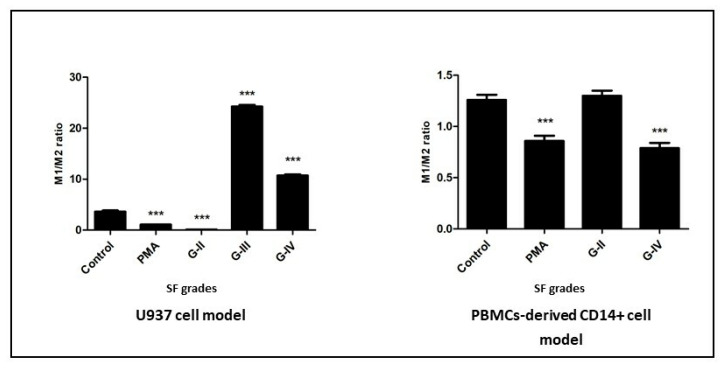
A grade-wise estimation of M1/M2 ratio in the newly differentiated cells after SF induction for 48 h in U937 and PBMC-derived CD14+ cells; in the assay on U937, the differentiation status was assessed using CD14, CD86, and CD163, while the assessment anti-body panel for PBMC-derived CD14+ cells included CD11C, CD86 (both M1 type), and CD163 (M2 type); M1/M2 ratio was estimated by dividing the average value of all M1 markers (U937—CD86 and PBMC-derived CD14 cells—CD11C + CD86) by the average value of the M2 marker (CD163) in each KL grade. *** *p* < 0.001, as compared to control.

In the assays on SF-induced U937 and PBMC-derived CD14+ cells to calculate M1/M2 ratio, the average percentage of all M1+ cells (in U937—CD86; in PBMC-derived CD14+ cells—CD11C + CD86) was divided by the average percentage of M2+ cells (CD163) in each KL grade. In the U937 cell model, the ratio was significantly higher in the cells induced with KL grade III and grade IV SF samples as compared to control. On the contrary, a significantly lower M1/M2 ratio was found in the cells treated with KL grade II SF samples (Figure 6).

In the PBMC-derived CD14+ cell model, M1/M2 ratio did not reveal any significant findings, possibly due to the lower percentage of polarized cells; in fact, a lower M1/M2 ratio was estimated in the cells induced with KL grade IV SF samples when compared to untreated cells (Figure 6).

### 3.7. Proteome Analysis of SF Samples

A grade-wise proteome analysis of SF samples was performed to identify the factors which can contribute to driving macrophage differentiation and polarization. Table 1 lists the proteins that are differentially expressed in SF samples along with their fold-change expression against the control group (SF samples of KL grade I) and their action on macrophages. This list includes proteins like macrophage migration inhibitory factor (MIF), macrophage-capping protein (CAPG/MCP), grancalcin, osteopontin C, and tumor necrosis factor alpha-induced protein-8 (TNFAIP8) as significantly expressed in different grades of OA SFs. Among these proteins, a progressive downregulation in MIF levels was seen. In contrast, CAPG/MCP, grancalcin, osteopontin, and TNFAIP8 revealed a significant elevation in KL grade III and IV samples and are associated with functions like macrophage migration, activation, polarization, and generating an inflammatory response, as specifically indicated in Table 1. The proteome analysis also revealed significant levels of many Ras-related RAB proteins in KL grade III and IV SF samples, which are cargo proteins and involved in endocytosis and vesicle transport.

## 4. Discussion

The concept of ‘niche’ represents a tissue-specific microenvironment to nurture monocytes/macrophages to be functionally imprinted by providing a physical scaffold for anchoring and survival factors, as elaborately explained by Guilliams and Scott, 2017 and Guilliams et al., 2020 [28,29]. Although in steady-state conditions, monocytes are not the only precursor cells of tissue macrophages [30], inflammatory conditions often facilitate a recruitment of circulatory monocytes to occupy the vacant site in response to the ‘macrophage disappearance reaction’, the phenomenon of a partial depletion of tissue-resident macrophages. The recruited monocytes further adopt a tissue-specific identity and take up a role similar to resident macrophages [31]. Likewise, in OA, which is a state of chronic low-grade inflammation, circulatory monocytes enter the affected joints and infiltrate the synovium to expand the population of resident macrophages, wherein a niche is required for a functional adaptation of these monocytes. Although the exact nature of the niche is still unclear, a common understanding in this regard is that this functional adaptation and macrophage differentiation and polarization are highly regulated by cues in the tissue microenvironment including cytokines, growth factors, and damage-associated molecular patterns of the extracellular matrix. These factors in the tissue microenvironment are thought to educate the recruited monocytes/macrophages about their phenotype and functions in the physiological or pathological context [30].

In the present study, the authors hypothesize that OA SF can act as a niche by providing the needed microenvironment for macrophage polarization in OA joints. The hypothesis is based on previous learnings that OA SF holds a cocktail of proinflammatory factors, cell degradation products, and immunoglobulins and can stimulate proliferative changes in various joint cell types. In support, the authors previously showed that the protein make-up of OA SF of different grades can induce cellular inflammation [9,10,32]. The present work is a continuation of these published studies to investigate whether the induced inflammation can further stimulate macrophage polarization, which is primarily responsible for the inflammation in OA joints. For this, an in vitro cell differentiation assay was designed, wherein U937 and PBMC-derived CD14+ cells were treated as macrophage precursors and incubated with OA SFs of different disease severity for 48 h. The assay design was based on the knowledge that macrophage polarization in in vitro conditions can be achieved by exposing the cells to different inducing agents, like interferon-γ (IFN-γ), lipopolysaccharide (LPS), PMA, IL-1β, and TNF-α to develop proinflammatory macrophages [33,34,35,36,37,38]. To our knowledge this is the first report wherein the pathobiological protein milieu of the diseased SF was used as a macrophage-polarization-inducing agent.

After 48 h of SF induction with different KL grades, U937 cells were found to be differentiated into CD14+, CD86+, and CD163+ phenotypes, while PBMC-derived CD14+ cells were turned into CD11C+, CD86+, and CD163+ phenotypes (Figure 2 and Figure 4). In the selected antibody panel, CD163 denotes M2 macrophages, while CD86 and CD11C represent the M1 macrophage phenotype; CD14 is a pan-macrophage marker. A grade-wise SF induction pattern showed an increase in the M2 phenotype after the induction with early OA samples (KL grade II), as was evident by an elevation in the percentage of CD163+ phenotype in both cell types (Figure 2D and Figure 4D). This phenotype elevation was significant in the case of U937 cells (Figure 2D). The outcome has many aspects—as said earlier, in vitro human macrophage polarization is reported to be influenced by sequential changes in tissue microenvironment. Therefore, a significant increase in CD163+ following the treatment with SF of KL grade II was possibly a natural combating response to a high degree of proinflammatory milieu in the KL grade II SF samples that was seen to decline in the later stages. The SF induction with KL grade III and IV showed a decline in the M2 phenotype and a simultaneous significant increase in CD86+ cells in the U937 assay (Figure 2). In our previous study, SF samples of KL grade II and III were able to generate the highest cellular inflammation and hence can be considered to have higher levels proinflammatory factors [9]

Despite of a higher proinflammatory milieu, a decline in the CD163+ phenotype after KL grade III SF induction can be attributed to the insufficiency of the combating efforts to neutralize the increasingly inflammatory microenvironment. Additionally, a possibility of phenotype switching in polarized macrophages has been suggested [39,40,41,42]. Accounting for these studies and OA SF as the inducing agent holding a stage-specific protein milieu, there is a scope for the inference that similar switching from the M2 phenotype to the M1 phenotype had taken place. In fact, a similar kind of phenotype switching is reported in chronic metabolic disorders like obesity, type-2 diabetes, and atherosclerosis [43,44]. Of note, obesity and type-2 diabetes are well-known confounding factors of OA [45,46]. Therefore, the possibility of switching the macrophage phenotype holds strong especially in OA joints, wherein a persistent low-grade inflammatory microenvironment is maintained. In this regard, the authors acknowledge that the present study is a pilot in nature, and they did not show actual switching of the macrophage phenotypes. The SF induction with KL grade III and IV showed a comparable stained cell population in the U937 assay, which denotes a phenotype shift in transit in OA progression from KL grade III to IV. This thinking is supported by comparable proinflammatory protein levels in KL grade III and IV SF samples (used for the induction), as revealed in grade-wise proteome analysis of OA SF (a subset of this data is shown in Table 1). The functional viability of the newly differentiated cells in the U937 assay in terms of cytokine production corelated with the staining pattern in the flow cytometry experiment (Figure 3).

In the cell differentiation pattern in the PBMC-derived CD14+ cells, all the selected cell surface markers showed a similar trend; this means that the highest percentage of CD11C, CD86, and CD163 was found after SF induction with KL grade II samples. The staining pattern of the CD163+ cells was similar in both cell types. However, in contrast to the assay on the U937 cells, a decline in the percentage of the M1 phenotype (CD11C+ and CD86+) was noted and can be attributed to a possible total cartilage wear-out effect in the terminal OA SF samples, ultimately causing a reduction in the associated inflammation. The two distinct categories of biomarkers in KL grade IV SF samples, their inflammation induction pattern, and correlation with cartilage loss are elaborately discussed in our previous publications [9,10,14]. Functional analysis of the newly differentiated cells in the assay on PBMC-derived CD14+ cells did not reveal any significant changes in the cytokine levels. In all, in vitro cell differentiation assays in both the cell types unanimously highlight the potential of OA SF to initialize monocyte/macrophage differentiation and polarization. The functional acquisition in the newly differentiated cells was more prominent in the U937 assay.

In the grade-wise SF proteome analysis, many macrophage-related proteins were seen as differentially expressed. For example, MIF was observed to be progressively downregulated across the KL grades. This protein is an immunoregulatory cytokine that arrests random immune cell movement, regulates cell recruitment, and is also responsible for M1/M2 polarization. In a xenograft model of glioblastoma, the downregulation of MIF led to the increased density of macrophages at the edge of the tumor [47]. In the current context, MIF may be responsible for the maintenance of the macrophage population in the joint tissues. A significant increase in CAPG/MCP (in the present study—KL grade III—38.62; KL grade IV—41.40) was recently shown to influence the migration behavior of macrophages by modulating actin dynamics through capping the growing end of actin filaments [48]. Grancalcin is a calcium-binding protein found to be increased by 42-fold in KL grade III and IV SF samples; this protein is involved in controlling calcium influx and modulates secondary signaling in the effector cell. Many RAS-related proteins were found to be significantly upregulated, especially in KL grade III and IV SF samples; these proteins are involved in various functions of macrophages, such as ER trafficking and phagocytosis. Members of RAB family proteins are low-molecular-mass monomeric GTPases localized on the cytoplasmic side of membranes; these proteins are involved in various functions of macrophages, such as ER trafficking and secretory pathways, and are essential for vesicular transport [49]. Osteopontin (showed 35.96-fold expression in KL grade IV), is a multifunctional protein involved in various signaling pathways. A recent publication by Liu et al. 2022 proposed that osteopontin affects macrophage polarization by promoting endocytic activity, but not inflammation [21]. As a member of the tumor necrosis factor alpha inducing protein family, TNFAIP8 plays role in maintaining immune homeostasis and inflammatory response. Silencing the TNFAIP8 protein using siRNA dramatically decreased IL-1β secretion in RAW264.7 macrophages [50].

A positive correlation between activated macrophages and OA severity is reported by Kraus et al., 2016, providing the first direct in vivo evidence for microphage involvement in OA pathology [51]. The status of the activated macrophages was determined in terms of M1 and M2 subtypes in OA SF samples by Liu et al., 2018 [7]. This study showed that the M1/M2 ratio of macrophages was significantly higher in knee OA patients when compared to healthy controls and correlated with OA severity. On similar lines, an estimation of the M1/M2 ratio in the newly differentiated cells in our study can serve as a functional validation of OA SF’s capacity to stimulate monocyte/macrophage differentiation. In the U937 cell assay, the M1/M2 ratio in the newly differentiated cells was significantly higher in the cells induced with KL grade III and IV SF samples as compared to untreated cells and correlated with OA severity (Figure 6). The ratio estimation, however, did not reveal any significant observations in the PBMC-derived CD14+ cell model, possibly because of a mixed cell population and lesser percentage of polarized macrophages as compared to the U937 cell model. The higher M1/M2 ratio after the SF treatment therefore underscored its potential to induce M1-dominated macrophage polarization. Given the knowledge that M1-dominated polarization of macrophages has anti-chondrogenic effects and leads to an increased production of proteolytic enzymes, including MMP-13 and ADAMTS5 [52,53], a significantly higher M1/M2 ratio as found in SF-induced cells can be a potential predictor of OA severity. Furthermore, the potential of the M1/M2 ratio in the induced cells to serve as a predictor of OA severity has the additional advantage of complementing the KL score system for better determination of OA severity. Accounting for the fact that there is a discrepancy between clinical and radiographic representations in OA, the KL score system, which is based on radiographic features, is not ideal for grading. Furthermore, an absence of a precise definition of joint space narrowing in this score system leads to an individual variation in the assessment [54]. Therefore, complementing the KL score system with a biochemical parameter like the M1/M2 ratio, which is a true cross-section of inflammatory pathology in OA, should be a welcome step. In fact, similar efforts have been made by the authors in their previously published study, wherein they reported a positive correlation between glycosaminoglycan (GAG) and the KL score system, underscoring its potential to complement this radiographic system [14]. A positive correlation of the M1/M2 ratio and GAG with the KL score also highlighted an existing relationship between the biochemical and radiographic progression in OA patients on the selected parameters.

Finally, some limitations should be taken into consideration while interpreting the results of this study. Firstly, some losses in storage-sensitive factors in SF must be attributed to the assays performed; however, we attempted to minimize it by limiting the SF sample processing time as described in the methodology. The percentage of differentiated immune cells after SF induction should be interpreted based on macrophage proportion, as evaluated in the SF cell immunophenotying experiment (Figure 1). The highest percentage of macrophages (estimated as CD14+ cells) was found in KL grade II fluid samples and was comparable to the other reports [55,56]. In these studies, the immunoprofiling of monocytes/macrophages in OA SFs was performed using a combination of surface markers, such as CD14, CD86, and CD163 or CD14 and CD16 markers. In our study, we used CD86 and CD163 markers as representative of the M1 and M2 type of macrophages, respectively, and the percentage of both the types in our SF samples was found to be negligible. The differentiation trends of U937 and PBMC-derived CD14+ cells were partially different, although the functional status of the newly differentiated cells was well matched to the respective differentiation pattern. Being a homogenous cell population, the outcomes on U937 cells were significant and more consistent, with a minimum degree of variability. On the other hand, the limited cell number and an obvious variation in the donor were typical obstacles encountered with the assay on PBMC-derived CD14+ cells. The authors did not aim to compare the differentiation trends in both of the cell types and want to emphasize that both of the cell types responded well to SF induction.

## 5. Conclusions

The present study demonstrated that OA SF can act as a niche for macrophage differentiation and facilitate the M1 type of polarization in the disease pathology by providing the necessary microenvironment; the cells are further responsible for implicating inflammatory and destructive responses in OA. Therefore, modulation of the SF niche could be key in controlling OA, and future research studies should be undertaken in this direction.

## Figures and Tables

**Table 1:** A grade-wise demonstration of key proteins found in the proteome analysis of OA SFs, which are linked with macrophage regulation.

No.	Protein	Action	Fold Change		
			G1-G2	G1-G3	G1-G4
1	Macrophage migration inhibitory factor (MIF)	Prevent random migration and stimulate population growth of macrophages	−0.295	−0.935	−38.678
2	Macrophage-capping protein, (CAPG/MCP)	Regulate cell migration through actin fiber modulation	0	38.623	41.396
3	Matrix metalloproteinase-9, (MMP9)	Multiple roles, including activation of macrophages	0	40.987	41.693
4	Prostaglandin E synthase 3, (PTGES3)	Regulate release of inflammatory prostaglandin by macrophages	37.965	37.109	0
5	Osteopontin C	Osteopontin affects macrophage polarization, promoting endocytic but not inflammation [21]	0	0	35.955
6	Grancalcin (GCA)	Control calcium influx and modulate secondary signaling	0	42.935	41.813
7	Tumor necrosis factor α-induced protein 8 (TNFAIP8)	Inflammatory response and immune homeostasis	0	35.575	38.093
8	Ras-related protein Rab-1A, RAB1A	Plays a role in cell adhesion and cell migration	0	40.627	39.556
9	Ras-related protein Rab-7a, RAB7A	Enables Phagocytosis	37.220	39.440	38.900
10	Ras-related protein Rab-10 RAB10	Phagosome maturation	0	38.686	38.171
11	Ras-related protein Rab-8B (RAB8B)	Antigen processing and presentation	1	39.618	37.869
12	Ras-related protein Rab-31 (RAB31)	Serves essential roles in vesicle and granule targeting [22]	0	37.347	37.264
13	Ras-related protein Rab-14 (RAB14)	Rab14 found to play a crucial role in phagocytosis, homotypic phagosome, and lysosome fusion [23]	0	37.731	37.122
14	Ras-related protein Rab-2A (RAB2A)	Ras-related GTP-binding proteins involved in the regulation of secretion [24]	0	39.676	36.786
15	Ras-related protein Rab-1B (RAB1B)	Regulates vesicular transport between the endoplasmic reticulum and successive Golgi compartments	0	35.756	35.921
16	Ras-related protein Rab-3D (RAB3D)	Involved in regulated exocytosis [25]	0	38.255	35.236
17	Ras-related protein Rab-5C (RAB5C)	Endocytosis [26]	36.733	38.253	35.156
18	Ras-related protein Rab-21 (RAB21)	Regulates integrin internalization and recycling; may regulate cell adhesion and migration [27]	0	39.135	0

## Data Availability

All datasets acquired in this study are included in the article. All the authors confirm that the presented data is factual and guarantee the validity of experimental results.

## Supplementary Materials

The following supporting information can be downloaded at: https://www.mdpi.com/article/10.3390/cells11244115/s1, Table S1: Patient demographic details from whom the SF samples were obtained and used during in vitro cell differentiation assay. Figure S1: Gating and cell sorting strategy to obtain CD14+ monocytes from PBMCs; for this, freshly isolated PBMCs were stained with CD14 (FITC) and physical parameters, forward scatter (FSC) and side scatter (SSC) were applied to select PBMCs population for sorting as shown in A; B and C represents the gating applied for CD14+ cell sorting.
