# Supplementary material for: Synovial Fluid in Knee Osteoarthritis Extends Proinflammatory Niche for Macrophage Polarization
Source: Cells. 2022 Dec 18;11(24):4115. doi: 10.3390/cells11244115 (PMC9776803; doi:10.3390/cells11244115)
Supplement: Supplementary file 1 [file cells-11-04115-s001.zip › cells-1966513-supplementary.pdf]

Table S1: Patient demographic details from whom the SF samples were obtained and used during *in vitro* cell differentiation assay

Patient No.	KL grade	Age (yrs)	Sex	BMI
P1	I	53	M	28.1
P2	I	40	F	28.1
P3	I	70	F	25.4
P4	I	65	M	27.5
P5	II	57	M	23.23
P6	II	42	M	31.1
P7	II	52	M	27.34
P8	II	63	F	22.41
P9	II	67	F	26.2
P10	III	31	M	25.21
P11	III	50	F	21.35
P12	III	55	F	20.3
P13	III	55	F	33.6
P14	III	50	F	18.5
P15	IV	75	F	24.63
P16	IV	59	M	21
P17	IV	70	M	23.2
P18	IV	75	M	19.81
P19	IV	59	M	27.1
P20	IV	77	F	25.5

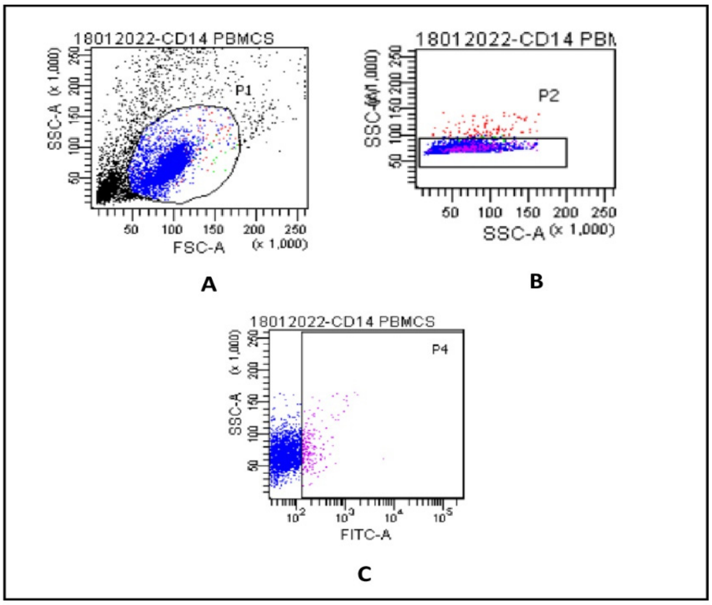

Figure S1: Gating and cell sorting strategy to obtain CD14+ monocytes from PBMCs; for this, freshly isolated PBMCs were stained with CD14 (FITC) and physical parameters, forward scatter (FSC) and side scatter (SSC) were applied to select PBMCs population for sorting as shown in **A**; **B** and **C** represents the gating applied for CD14+ cell sorting
